# Arm-Presentation in a Twin Case, Terminating by “Spontaneous Expulsion”

**Published:** 1884-10

**Authors:** 


### Arm-Presentation in a Twin Case, Terminating by "Spontaneous Expulsion."

Mr. Ford, in the *British Medical Journal*, reports the delivery of a full-grown child, presenting as follows: The left arm of the child was projecting from the vulva; the head was resting on the pubes; the left side of the thorax was over the outlet and the buttocks occupied the hollow of the sacrum. Pains were good; the child was dead, the first child having been delivered by a midwife three hours before, alive. The cross, birth was delivered without assistance by the accoucheur four and one-quarter hours after the first.

